# Community Susceptibility and Resiliency to COVID‐19 Across the Rural‐Urban Continuum in the United States

**DOI:** 10.1111/jrh.12477

**Published:** 2020-06-16

**Authors:** David J. Peters

**Affiliations:** ^1^ Department of Sociology Iowa State University Ames Iowa

**Keywords:** COVID‐19, community resiliency, county, rural‐urban, susceptibility

## Abstract

**Purpose:**

This study creates a COVID‐19 susceptibility scale at the county level, describes its components, and then assesses the health and socioeconomic resiliency of susceptible places across the rural‐urban continuum.

**Methods:**

Factor analysis grouped 11 indicators into 7 distinct susceptibility factors for 3,079 counties in the conterminous United States. Unconditional mean differences are assessed using a multivariate general linear model. Data from 2018 are primarily taken from the US Census Bureau and CDC.

**Results:**

About 33% of rural counties are highly susceptible to COVID‐19, driven by older and health‐compromised populations, and care facilities for the elderly. Major vulnerabilities in rural counties include fewer physicians, lack of mental health services, higher disability, and more uninsured. Poor Internet access limits telemedicine. Lack of social capital and social services may hinder local pandemic recovery. Meat processing facilities drive risk in micropolitan counties. Although metropolitan counties are less susceptible due to healthier and younger populations, about 6% are at risk due to community spread from dense populations. Metropolitan vulnerabilities include minorities at higher health and diabetes risk, language barriers, being a transportation hub that helps spread infection, and acute housing distress.

**Conclusions:**

There is an immediate need to know specific types of susceptibilities and vulnerabilities ahead of time to allow local and state health officials to plan and allocate resources accordingly. In rural areas it is essential to shelter‐in‐place vulnerable populations, whereas in large metropolitan areas general closure orders are needed to stop community spread. Pandemic response plans should address vulnerabilities.

The coronavirus disease 2019 (COVID‐19) global pandemic continues to be a major public health crisis in the United States, severely affecting the health and socioeconomic well‐being of many Americans.^1^ Serious complications from COVID‐19 have fallen heavily on people 65 years of age and older, accounting for 50% of hospital and ICU admissions, and 80% of deaths.^2^ Of those hospitalized with COVID‐19, 75% have some underlying medical condition regardless of age—typically diabetes, chronic lung, and cardiovascular diseases.^3^ Large metropolitan areas have garnered the most attention in academic and policy discussions about COVID‐19, due to large numbers of cases and deaths.^4^ Missing from the discussion is the pandemic's impact on rural America. This is an important omission as recent evidence shows rural places have higher rates of COVID‐19 comorbidity, making them more susceptible to the pandemic.^5^

However, current information provides an incomplete picture by an overreliance on state‐level aggregations and confirmed case counts. The former does not explore differences across rural and urban contexts at the county level, as has been done with other public health issues like the opioid crisis.^6^ The latter is a poor estimate of coronavirus exposure in the population because of test unavailability, laboratory delays, and rules on who is eligible to be tested.^7^ Case counts provide no information on the severity of the disease, such as how many are asymptomatic or hospitalized. In short, one should be extremely cautious in making state and county comparisons using only case counts as a measure of the pandemic.^8^ Rural places may still be susceptible to COVID‐19 even in the absence of confirmed cases. This makes rural places statistically invisible and creates a false sense of rural immunity, even as projections anticipate rising numbers of cases as the pandemic takes hold.^9^ For example, COVID‐19 outbreaks in rural meat packing communities caught public health and government officials off‐guard, disproportionally impacting Hispanic and other minority workers.^10^, ^11^

Knowing susceptibility is only part of the equation in this rapidly evolving crisis. Rural public health officials also need to know whether at‐risk communities can endure a potential outbreak of COVID‐19.^12^ Is the health care system equipped to handle an influx of cases? Are there socially vulnerable populations that may be indirectly impacted? How vulnerable is the local economy to government‐ordered closures? Is the community able to address the social and psychological repercussions of COVID‐19? In the social sciences, community resiliency is the term given for the ability of a place to cope with, adapt to, and recover from hazards like the current pandemic.^13^, ^14^ Previous research has linked resiliency with greater social capital, collective action, and robust governance structures.^15^, ^16^ Resilience in urban areas is primarily driven by economic capital, whereas social capital is more important in rural areas.^17^ In the public health literature, resiliency and social capital are argued to promote community compliance with social distancing and shelter‐in‐place orders,^18^ better health behaviors that make residents less susceptible to disease,^19^, ^20^ improved health care infrastructure and access to properly treat those with serious health issues,^21^ and better health outcomes for persons of color.^22^ A recent analysis in rural Australia used spatial methods to identify geographic areas having both COVID‐19 vulnerable populations and poor access to health services, which can be used in rural health planning.^23^ However, there is scant literature at present examining community resilience to influenza pandemics, especially COVID‐19.

The purpose of this analysis was to address current gaps in the public health literature by linking community susceptibility and resiliency to the current pandemic. First, a COVID‐19 susceptibility scale was created at the county level using indicators linked to serious complications of the disease, as presently known. Rates per population were used to measure relative susceptibility, instead of counts for absolute susceptibility. The former allows for better detection of COVID‐19 risks in smaller rural counties, while the latter would only identify large population centers that are at‐risk. Second, the potential resiliency or vulnerability of high‐susceptibility counties was assessed along a number of health, economic, and social indicators. This paper makes a unique contribution to the public health literature by measuring COVID‐19 risks and resiliency at the meso‐scale using extant data sources. By contrast, the small number of existing studies primarily focuses on individual psychological perceptions of the pandemic, and not on community susceptibility using objective indicators.^24^

## Methods

### Data

Units of analysis are N = 3,079 counties in the 48 conterminous United States based on 2000 Census geographies, with modifications.^i^ The 11 *susceptibility indicators* were taken from the US Census Bureau's American Community Survey (ACS)^25^ and County Business Patterns (CBP)^26^ and US Centers for Disease Control and Prevention's National Vital Statistics System (NVSS).^27^ Indictors were chosen based on presently known correlates of the disease.^2^, ^3^ Population measures from the 2014‐2018 ACS include: population density per square mile (measuring the potential for community spread), percent population living in group quarters or institutional settings (eg, college, correctional, or care facilities), percent population aged 65‐84 years, and percent aged 85 years and older. The presence and scale of nursing and elderly care facilities (NAICS 6231 and 6233) in the community were measured using employment per 10,000 people in 2016. Employment in meat processing facilities (NAICS 3116, less rendering firms) per 10,000 is also included to capture COVID‐19 risks in large packing communities.^11^ Both were estimated by the Upjohn Institute for Employment Research using CBP place‐of‐work establishment data.^ii^ Proxy measures of health‐compromised populations in 2016‐2018 were obtained from NVSS and include: age‐adjusted mortality rates per 100,000 from malignant neoplasms (C00‐97), diabetes mellitus (E10‐14), cardiovascular diseases (I00‐78), influenza and pneumonia (J10‐18), and chronic lower respiratory diseases (J40‐47). Mortality was reported by residence of the decedent and pooled over 3 years to provide more stable estimates.^28^ *Indicators of resiliency and vulnerability* from ACS include population structure and household characteristics, employment and income by place‐of‐residence, disability status, and health insurance coverage. The presence of health care, social services, and community organizations in the county is from CBP/Upjohn Institute. Other indicators include charitable contributions per capita in 2017 from the Internal Revenue Service^29^; property crimes per 100,000 between 2015 and 2017 from the Federal Bureau of Investigation^30^; and miles of United States interstate highways per square mile, calculated using GIS software.

### Statistical Procedures

Exploratory factor analysis (EFA) was used to construct the COVID‐19 susceptibility scale. Principal components extraction and varimax rotation were used to combine the 11 indicators into 7 distinct factors accounting for 81.5% of the original variance in the data. All assumptions of EFA were met, with low factor correlations supporting use of orthogonal rotation.^31^, ^32^ Since the purpose was scale construction, the number of factors was determined by maximizing explained variance and factor validity, instead of more typical criteria.^iii^ Most factors exhibited high factor loadings (*λ* > 0.8) and all indicators showed high communalities (*h* ^2^ >0.7), indicating a robust solution. Results of the EFA are presented in Table 1, with the scree plot in the Appendix (available online only). *Health‐compromised susceptibility* (factor 1) includes mortality from cancer, cardiovascular, chronic lower respiratory, and flu/pneumonia diseases. *Seniors and elders susceptibility* (factor 2) includes shares of people age 65‐84 and age 85 and older. *Care facilities and elders susceptibility* (factor 3) includes local employment in nursing and elderly care facilities, and a strong cross‐loading with residents 85 years and older. This indicates a strong correlation between care facilities and the very old. A number of single indicator factors measure susceptibility from *meat processing facilities* (factor 4), *population density* (factor 5), *group quarters* (factor 6), and *diabetes mortality* (factor 7). Single indicator factors were largely uncorrelated and independent of other variables. Factor scores follow a *z* distribution and were estimated using Thurstone's regression method to maximize score determinacy.^31^ The total susceptibility scale was created by censoring the factor scores at *z* ± 5 to limit the influence of extreme values, then summing the 7 components.

**Table 1:** COVID‐19 Susceptibility Scale Factor Analysis for N = 3,079 Counties in the Conterminous United States

	Factors							
	1	2	3	4	5	6	7	*h* ^2^
**Factor Loadings and Variance**								
Population density (sq. mi)	−0.06	−0.07	−0.01	−0.02	**0.99**	−0.02	−0.03	0.99
Group quarters (%)	0.09	−0.11	0.07	−0.05	−0.02	**0.96**	0.00	0.95
Age 65‐84 (%)	0.06	**0.88**	0.05	−0.08	−0.08	−0.13	−0.12	0.83
Age 85 and older (%)	−0.13	**0.67**	**0.59**	−0.01	−0.01	−0.01	0.01	0.81
Elderly and nursing care jobs (10k)^†^	0.01	0.10	**0.88**	0.08	0.00	0.08	0.05	0.80
Cancer mortality (100k)^†^	**0.78**	0.06	0.01	0.00	0.02	0.07	0.26	0.69
Cardiovascular mortality (100k)^†^	**0.82**	−0.08	0.00	0.06	0.02	0.04	0.05	0.68
Lower respiratory mortality (100k)^†^	**0.77**	0.06	−0.09	0.03	−0.11	0.04	0.10	0.63
Diabetes mortality (100k)^†^	0.27	−0.11	0.05	−0.02	−0.03	−0.01	**0.92**	0.94
Flu and pneumonia mortality (100k)^†^	**0.57**	−0.36	0.36	−0.22	−0.06	−0.21	−0.13	0.69
Meat processing jobs (10k)^†^	0.04	−0.07	0.07	**0.97**	−0.02	−0.05	−0.02	0.96
Eigenvalue	2.55	1.72	1.08	1.04	0.99	0.80	0.78	n.a.
Variance explained (%)	23.19	15.64	9.83	9.42	9.01	7.28	7.10	n.a.
**Parallel Test**								
Random eigenvalue	0.88	0.86	0.83	0.82	0.81	0.80	0.79	n.a.
Parallel test (model–random eigenvalue)	1.67	0.86	0.25	0.21	0.18	0.01	−0.01	n.a.

Notes: † = rate per population; bold indicates high factor loading. Principal components extraction; varimax rotation; h2 = communality or variance explained by factors. Sampling adequacy KMO = 0.67; root mean square residual RMSR = 0.07.

Next, a multivariate general linear model (traditionally MANOVA) was used to estimate unconditional mean differences across a number of resiliency indicators using the Games‐Howell test, which is robust to unequal group sizes and variances.^33^ To explore differences across the rural‐urban continuum, a modified version of 2003 urban influence codes from the US Department of Agriculture was used to delineate 5 classes: large metropolitan areas containing a city of 1 million or more people; midsize metro areas with a city of 250,000 to 999,999; small metros areas with a city of 50,000 to 249,999; micropolitan areas with a city of 10,000 to 49,999; semirural counties that include a town of 2,500 to 9,999 residents; and completely rural counties with no town over 2,500 people.^iv^ Descriptive statistics of the susceptibility indicators by rural‐urban classes is presented in the Appendix (available online only).

## Results

### COVID‐19 Susceptibility

**Figure 1:**
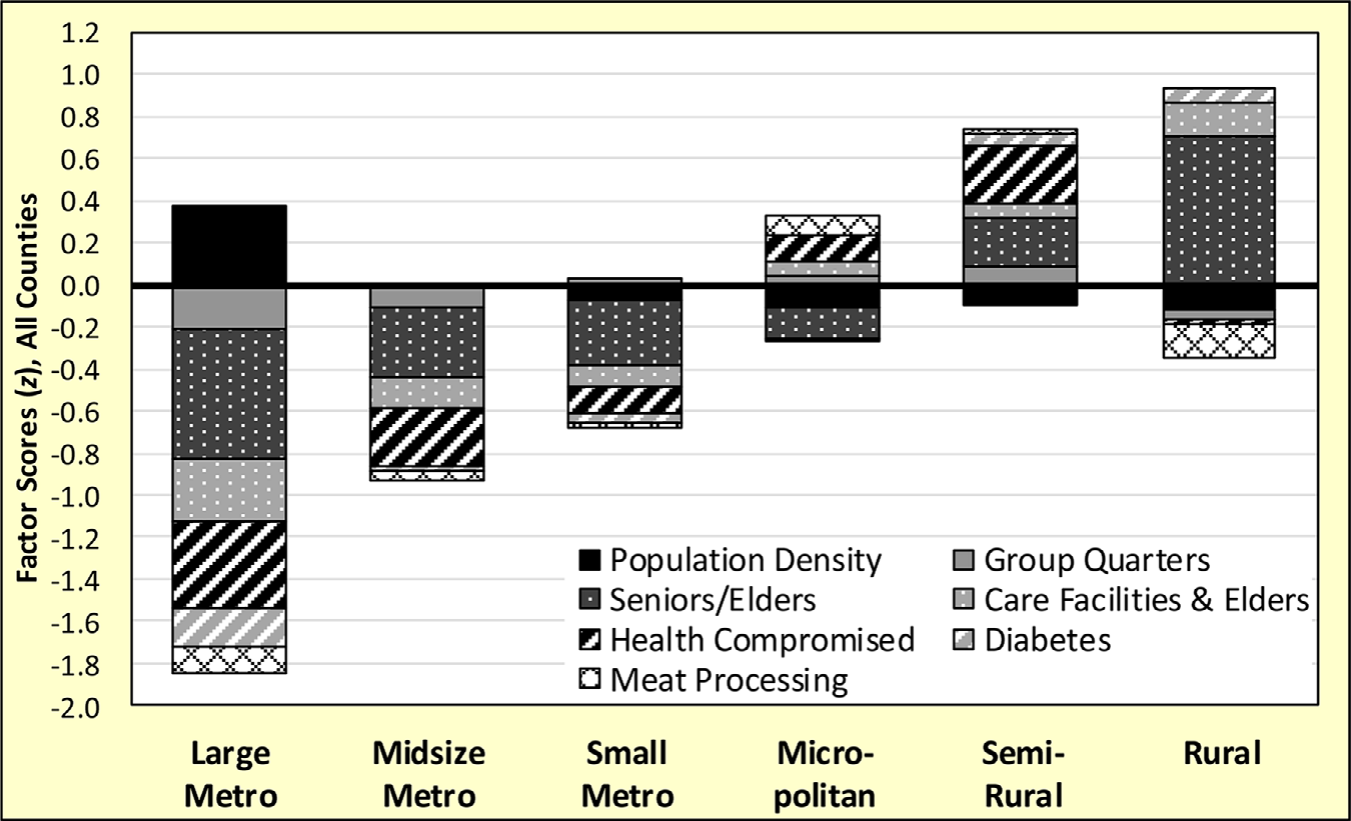
COVID‐19 Susceptibility Factor *z*‐Scores for N = 3,079 Counties in the Conterminous United States.

**Figure 2:**
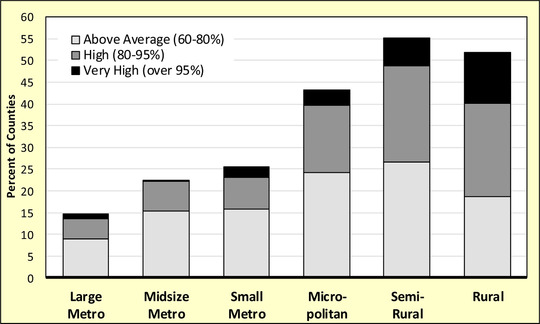
Share of Counties Susceptible to COVID‐19 by Percentiles for N = 3,079 Counties in the Conterminous United States.

**Figure 3:**
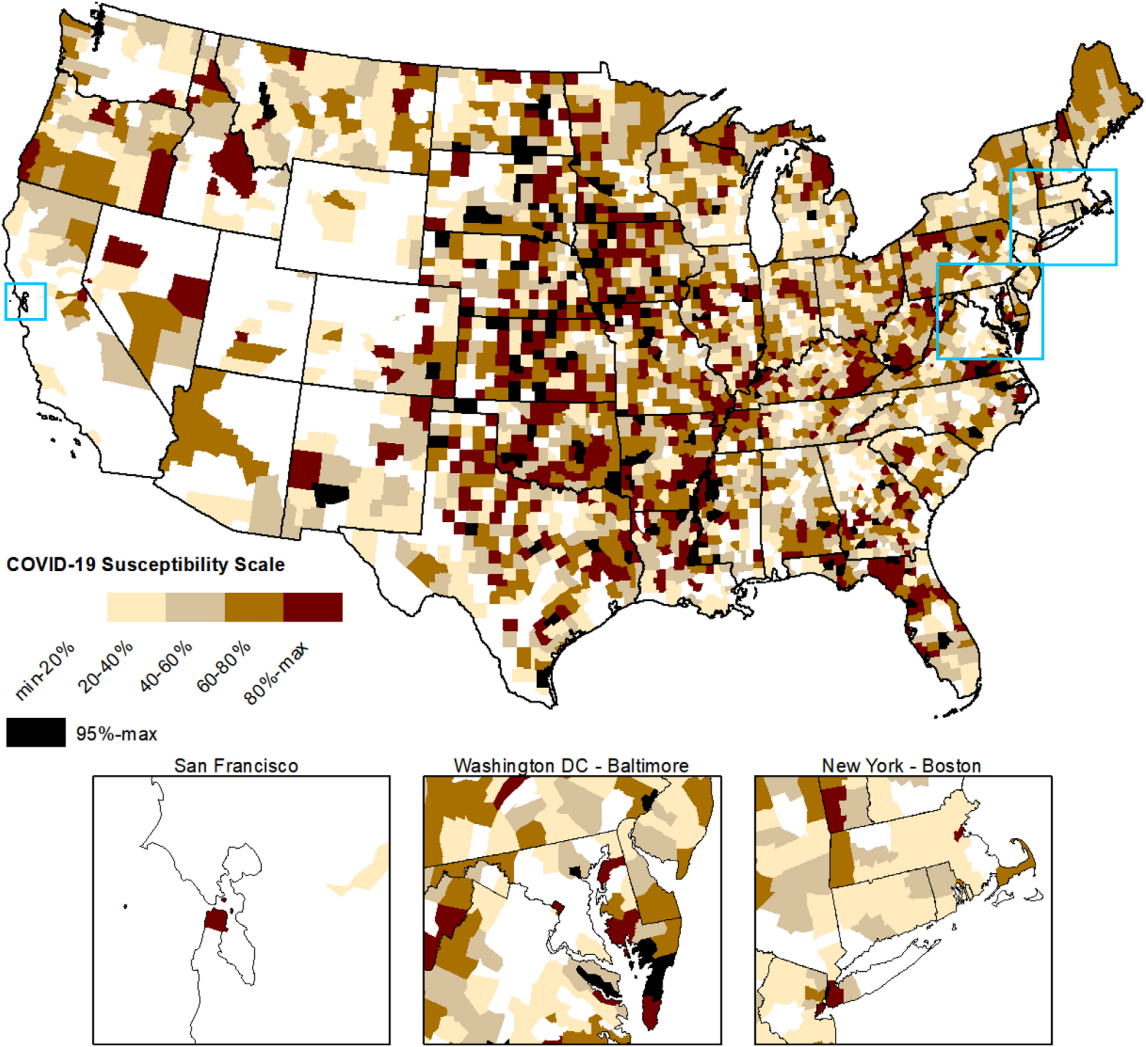
Spatial Distribution of COVID‐19 Susceptibility Scores by Quintiles for N = 3,079 Counties in the Conterminous United States.

Several interesting patterns emerge from susceptibility scores when disaggregated across the rural‐urban continuum. Nonmetropolitan counties, including micropolitan and rural, are more susceptible to COVID‐19 than metropolitan ones. In Figure 1, the risk of serious complications increases as one progresses from large and midsize metropolitan counties (*z* < −1.0) to more semirural and rural ones (*z* >0.5). Large shares of non‐metropolitan counties are at above average or high (fourth and fifth quintiles) susceptibility to COVID‐19 complications, as evidenced by Figure 2. About 33% of rural counties fall into the high‐risk group, as do 29% of semirural and 19% of micropolitan places. The spatial distribution of susceptibility scores by county is presented in Figure 3, where high‐risk communities are concentrated in the Great Plains, Midwest, some Great Lakes states, and in the lower Mississippi Delta. High susceptibility is also found in densely populated metros including major cities from Boston to Washington, DC on the East Coast, and San Francisco to the West.

Rural counties are primarily susceptible from large senior and elder populations, and COVID‐19 outbreaks are likely to originate in care facilities for the elderly due to their presence in these aging counties (see Figure 1). Care facilities pose risks for residents and workers alike. Despite being rural, lack of large meat processing facilities lowers susceptibility, likely caused by a small labor force. In semirural places, severe cases of COVID‐19 are likely to be caused by a mix of older residents, health‐compromised individuals, and people living in institutional settings. By having a town over 2,500, semirural counties typically serve as regional trade centers providing health care and education services for surrounding counties, likely attracting residents who fall into at‐risk subpopulations that increase community susceptibility. Micropolitans are at above average susceptibility due to health issues, large numbers of meat processing workers, and care facilities for the very old. Despite the presence of elder care facilities, the relatively small senior/elder population lowers susceptibility overall. Elder care and meat packing facilities tend to locate in larger, hence younger, counties with adequate labor and supporting services.

By contrast, metropolitan counties are at much lower susceptibility due to younger populations and better health outcomes. In all size classes, metros have fewer shares of seniors/elders, fewer care facilities for the elderly, lower mortality from diseases that make people vulnerable to COVID‐19, and fewer shares of people living in institutions. Despite these advantages, large metros of a million or more people are susceptible to community spread due to high population densities. About 6% of the nation's largest metro counties fall into the top quintile of susceptibility scores, including major cities in the Northeast that are the current epicenters of the pandemic. This shows that a handful of high‐susceptibility and high‐population metros can be the locus of numerically large outbreaks, driving national cases and deaths. This suggests certain large cities will always be susceptible to viral pandemics due to dense living conditions, even though they have relatively healthy and younger populations.

### COVID‐19 Resiliency

**Figure 4:**
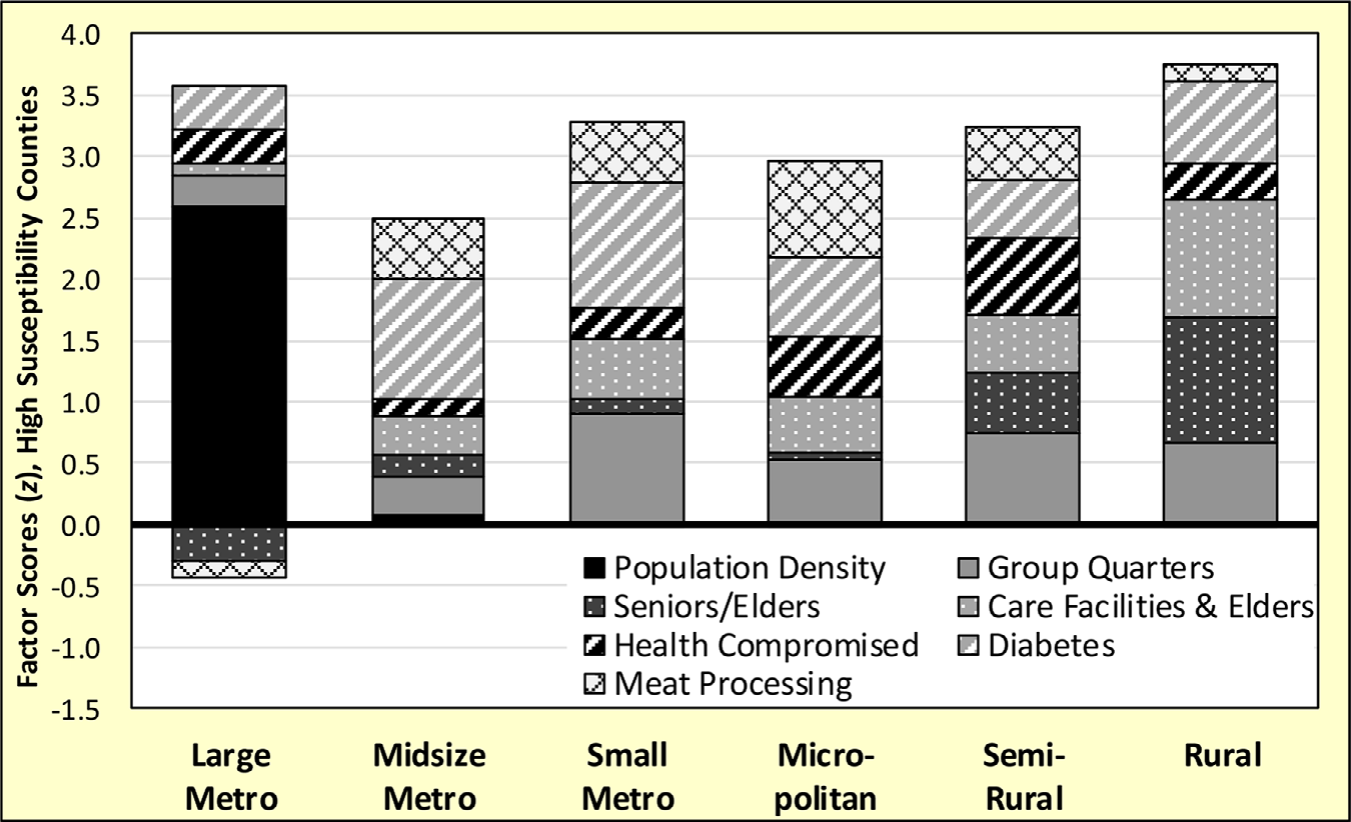
COVID‐19 High Susceptibility (80th Percentile or Higher) Factor *z*‐Score Estimates for N = 616 Counties in the Conterminous United States.

The second objective of this paper is to assess the resiliency or vulnerability of counties at high susceptibility to COVID‐19, defined as places with scores in the 80th percentile or higher. Although scores are generally similar across the rural‐urban continuum, the components of susceptibility are quite different (see Figure 4). In the most rural counties, susceptibility is driven by large shares of seniors/elders, care facilities for those over age 85, diabetes, and group quartered residents. There is also elevated risk from health‐compromised people. Larger semirural communities are susceptible from large institutional populations, people with health and diabetes issues, and meat processing facilities. Seniors, elders, and care facilities are secondary contributors, being smaller risk components than in more rural counties. COVID‐19 outbreaks in micropolitan and small metro counties are likely to occur in large animal slaughter and meat processing facilities, affecting both workers and residents. Given many workers are minorities, this may account for high rates of diabetes in these meat packing communities.^22^ In general, susceptibility in smaller cities and counties tends to be driven by health‐compromised persons, older residents, meat packing, and institutions like colleges, corrections, and care facilities. By contrast, large metropolitan cities have relatively healthier and younger populations. What drives susceptibility in the largest counties is population density, where a small number of COVID‐19 cases can spread rapidly due to close contact environments.

Now that high susceptibility counties have been identified, are their social systems resilient enough to withstand an outbreak should it occur? To answer this question, socioeconomic and health indicators were compared across the rural‐urban continuum, with the results presented in Table 2. Rural and semirural places are most vulnerable when it comes to health status, having fewer physician offices, more people with a self‐care disability, and more residents without any private or public health insurance. Not only are disabled persons more susceptible to COVID‐19, but infections among care providers may limit essential services to this at‐risk population. Lack of health insurance may cause people to avoid seeking immediate treatment, worsening their condition and increasing risk of community spread. Financial stress may also occur if the uninsured are not able to pay medical bills. Rural places also have lower access to mental health (including substance abuse) and family social services to address the emotional and financial stress caused by the pandemic. On the other hand, susceptible rural places do not appear to be disadvantaged to most urban ones (except in the largest metros) in terms of per capita hospital and pharmacy staffing. National standards and certifications likely minimize staffing differences across the rural‐urban continuum. In terms of population characteristics, the major vulnerability is lack of broadband specifically and any Internet access generally. This limits the ability of residents to take advantage of telemedicine services for the sick, telecommuting to prevent layoffs, and distance education for youth. Rural places are also vulnerable because they lack access to the interstate system, making transportation of patients, health providers, and supplies difficult.

**Table 2:** Resiliency Indicators in 2018 for N = 616 Counties at High Susceptibility (80th Percentile or Higher) to COVID‐19 in the Conterminous United States

	Metropolitan			Nonmetropolitan		
	(a)	(b)	(c)	(d)	(e)	
	Large Metro	Midsize Metro	Small Metro	Micropolitan	Semirural	Rural
	N = 23	N = 23	N = 33	N = 126	N = 230	N = 181
**Population**						
Population in 1,000s (#)	708.53	104.69^a^	48.84^a^	36.57^ab^	15.70^abcd^	7.13^abcde^
Age 17 and younger (%)	20.43	21.79	21.30	22.49^a^	21.47^d^	20.92^d^
Minority population (%)	50.72	29.23^a^	25.09^a^	27.55^a^	23.11^a^	16.36^abcde^
Single‐head families, children (%)	42.68	36.77	34.91^a^	38.34	35.01^ad^	30.17^abcde^
In migration, all origin (%)	7.52	7.92	7.95	6.58^c^	7.05	6.75
In migration, international (%)	0.70	0.33^a^	0.23^a^	0.34^a^	0.26^a^	0.23^a^
Limited English ability (%)	5.34	1.38^a^	1.63^a^	2.35^a^	1.35^ad^	0.88^ade^
No broadband internet (%)	38.97	47.17^a^	48.32^a^	49.00^a^	54.22^abcd^	50.34^ae^
No internet service (%)	20.35	24.84^a^	24.95^a^	25.33^a^	27.84^acd^	27.83^acd^
Interstate density (mi^2^*100)	16.06	3.93^a^	2.05^a^	1.64^ab^	1.02^abd^	0.46^abcde^
**Health Status**						
Retail pharmacy capacity (10k)^†^	29.55	18.04^a^	16.86^a^	22.16^ac^	20.33^a^	21.30^a^
Physician capacity(10k)^†^	73.97	50.56	58.75	50.04^a^	25.36^abcd^	20.37^abcd^
Hospital capacity (10k)^†^	263.50	123.39^a^	133.75^a^	159.17^a^	143.33^a^	132.31^a^
Mental health capacity (10k)^†^	27.66	14.38	13.96	16.90	20.31	4.22^abde^
Family social srvs. capacity (10k)^†^	73.71	38.72	29.52^a^	50.05^c^	31.43^ad^	31.29^ad^
Community social srvs. capacity (10k)^†^	12.19	3.38^a^	8.02	7.73	3.08^a^	6.00^a^
Self‐care disability (%)	3.15	3.44	3.48	3.41	4.52^abd^	3.99^abd^
Independent living disability (%)	7.10	7.72	7.30	7.50	7.75	7.39
No health insurance (%)	8.96	9.54	10.61	9.96	12.89^abd^	12.86^abd^
**Employment and Income**						
Labor force participation (%)	45.04	41.59	40.92^a^	41.19^a^	40.52^a^	40.89^a^
Agriculture, forestry and fishing (%)	0.93	3.16^a^	3.85^a^	4.03^a^	6.10^abcd^	10.77^abcde^
Manufacturing and construction (%)	13.60	20.27^a^	18.96^a^	21.17^a^	20.72^a^	17.14^abde^
Transportation and warehousing (%)	5.07	4.01^a^	4.06^a^	4.07^a^	4.32^a^	4.34
Retail, leisure, and personal srvs. (%)	26.47	24.50	24.54	24.61	23.27^ad^	21.56^abcde^
Professional and business srvs. (%)	18.44	12.48^a^	11.87^a^	9.40^abc^	9.13^abc^	8.38^abcde^
Health and social assistance srvs. (%)	15.20	15.00	14.82	15.38	14.41^d^	14.74

	High‐Risk Metropolitan			High‐Risk Nonmetropolitan		
	(a)	(b)	(c)	(d)	(e)	
	Large Metro	Midsize Metro	Small Metro	Micropolitan	Semirural	Rural
	N = 23	N = 23	N = 33	N = 126	N = 230	N = 181
Median HH income ($)	55,061	48,099	47,721	44,324^ac^	43,741^abc^	44,440^ac^
Poverty (%)	18.72	16.68	16.84	18.81	17.77	16.71^d^
Income inequality (Gini)	48.44	46.07^a^	44.67^a^	45.16^a^	45.25^a^	44.80^a^
Median home value over income (#)	4.99	2.55^a^	2.42^a^	2.42^a^	2.26^abd^	2.10^abcde^
**Social**						
Religious org. capacity (10k)^†^	45.41	49.03	46.55	50.01	48.05	43.97^d^
Foundation org. capacity (10k)^†^	18.36	1.64^a^	1.68^a^	3.95	0.84^a^	1.06^a^
Social advocacy org. capacity (10k)^†^	20.55	2.26	1.24	2.61^c^	1.72	1.13^d^
Civic & social org. capacity (10k)^†^	12.61	3.59^a^	5.52^a^	8.64^b^	6.32	5.66^ad^
Work‐related org. capacity (10k)^†^	32.92	4.09	7.63	5.05	4.00^c^	2.56^cde^
Charitable contributions ($/person)	819.09	499.63	375.17^a^	341.36^a^	303.49^abd^	260.97^abcde^
Property crime rate (per 100k)	579.69	649.74	517.71	698.39	397.19^bd^	186.19^abcde^

Notes: † = capacity is establishment employment per 10,000 residents. Games‐Howell mean difference test; two‐tailed at *P* <.05. Difference from: a = large metro (city of 1 million or more); b = midsize metro (city of 250k‐999k); c = small metro (city of 50k‐249k); d = micropolitan (city of 10k‐49k); e = semirural (town of 2.5k‐9k). Rural does not have a town of 2,500 or more residents.

Looking at the social capacity to respond to the pandemic, rural places are vulnerable by having low rates of charitable giving, fewer work‐related organizations (eg, business, professional, and labor associations), and fewer community civic organizations. This limits the ability of susceptible rural communities to respond immediately using local resources, forcing them to wait for state and federal assistance. Local efforts to provide services to vulnerable residents, to help the unemployed, and to resurrect local businesses will be hampered by lack of funds and leadership that community organizations provide. Coupled with low Internet access, rural labor markets provide few opportunities for telecommuting. Although median incomes are lower, likely due to cost of living differences, poverty rates are remarkably similar across most rural‐urban categories. This indicates that susceptible rural places are no poorer in relative terms than metro cities.

On the other hand, rural communities are more resilient to the near‐term effects of the pandemic by having an employment base less affected by falling demand and government‐ordered closures.^34^ Fewer rural residents work in retail trade, leisure, and personal services that have experienced closures and layoffs. Production agriculture, an important part of the rural economy, has been minimally impacted thus far. However, labor‐intensive fruit and vegetable farms in the western United States are vulnerable to outbreaks among farm laborers.^35^ In most rural places the goods‐producing sector is small, but semirural communities have larger employment shares making them more vulnerable to infection from close working conditions, and more prone to layoffs from reduced demand. Rural households are at low risk of housing distress, as the ratio of median home value to income is small. Lastly, property crime rates are very low in rural and semirural communities. This suggests public order will be maintained should health or economic conditions caused by the pandemic worsen to the point of crisis.

Micropolitans are generally more resilient than rural places by having better Internet access, more physician offices, fewer disabled persons, fewer uninsured, more family social service organizations to deal with stress, and greater social capital in terms of giving and civic groups. However, micropolitans are still more vulnerable than metropolitans on most of these indicators. Micropolitans are more economically vulnerable than their rural peers as more residents work in layoff‐prone sectors like manufacturing, construction, retail, and leisure services. Property crime is already a major problem, and there is a real risk of widespread theft and vandalism if the pandemic causes severe food or health care shortages.

The largest metropolitan counties have a distinct set of advantages and disadvantages related to COVID‐19. Large cities are more vulnerable because of ethnoracial diversity, having large shares of minorities, more limited English speakers, and higher international migration. Some minority groups are more susceptible to certain inequality‐driven health complications related to COVID‐19, especially among African Americans that comprise a sizable share of the minority population in the largest metros.^36^ Communicating public health information is complicated by having a diversity of languages. Having more single‐headed families is a concern if the parent is sick and has no other person to care for their children, potentially straining local social services. Economically, metros are more vulnerable to layoffs as more residents are employed in retail trade and leisure services, most having been closed by government orders. American cities are highly connected to the interstate system and many residents are employed in the transportation sector, increasing the likelihood of COVID‐19 entering from distant locations at home or abroad. While jobs in transportation and warehousing are not prone to layoffs, handling and delivery requires contact with many people across varied locations, increasing the risk of COVID‐19 infection and community spread. The most concerning aftershock of the pandemic is that metro residents are very vulnerable to severe housing distress due to high housing costs, as home values are 5 times median incomes. Even a temporary loss of income would quickly drain savings, potentially leading to widespread foreclosures and downturn in the local economy. Despite high median incomes, high income inequality means it is concentrated among a few top earners and not among all households.

However, large metros are also resilient in a number of ways. They have well‐developed social service and mental health systems to deal with pandemic‐related stress, they have fewer disabled residents who cannot care for themselves, and they have fewer shares of uninsured individuals. In addition, high staffing levels in urban hospitals indicate they have the capacity and expertise to deal with severe complications of COVID‐19. The economy is generally robust, with high labor force participation rates and high median household incomes, although the latter likely reflects higher costs of living. A large share of residents will be able to continue working in their professional and business services jobs by telecommuting from home. This is facilitated by widespread access to broadband and other Internet services. In terms of social capacity, large metros are well resourced by private donations as evidenced by high charitable giving and large community foundations. Private funds give large cities the independence and flexibility to identity and address needs as they see fit, unlike government funds that often have stipulations.

## Discussion

There is an immediate need to assess the susceptibility of serious COVID‐19 complications and a community's socioeconomic resiliency to these complications before such cases become widespread, especially in rural areas. Knowing specific types of susceptibilities and vulnerabilities ahead of time allows local and state health officials to plan and allocate resources accordingly. In rural and micropolitan communities, it is essential to disperse or shelter‐in‐place specific vulnerable populations quickly. Where possible, this includes the health‐compromised, senior citizens, people living in institutional settings, and workers in large meat processing facilities. To accomplish this, local agencies and providers need to focus their efforts at providing essential services (eg, food, health care, and personal care) to these home‐ or facility‐bound groups. The monumental task of providing these services to a dispersed population will require volunteers and community groups as partners, forcing rural places to fall back on their community's social capital. Being sparsely populated, the risk of community spread is perhaps less of a concern, suggesting general shelter‐in‐place orders in rural communities may be less effective than targeted ones protecting the most vulnerable. However, in large metropolitan cities susceptibility is clearly driven by high population densities, making strict business closure and shelter‐in‐place orders essential to slow community spread of COVID‐19.

Lack of health care and social services makes rural communities particularly vulnerable. Any state or national response to the pandemic will be hindered by logistical barriers deploying health providers and supplies over a large geographic area.^23^ Lack of broadband access means health planners cannot rely solely on telemedicine to fill the gaps in rural areas; it also means medical boots on the ground to provide care. Coordinating efforts among many rural hospitals and local jurisdictions presents organizational barriers that may limit effective responses.^37^ It is essential that each state reexamine its rural pandemic response plans to clarify lines of authority between jurisdictions, identify primary regional hospitals, stockpile needed medical supplies, and detail how health professional shortages will be addressed. For the latter, a current registry of former or retired health providers in rural areas should be maintained, including contact information, to address staff shortages quickly. Similar response plans are also needed to address the emotional and socioeconomic harm once the health crisis has passed. Counseling services to deal with grief, stress, family issues, and financial problems are essential if rural families and communities are to fully recover from COVID‐19 and similar pandemics in the future. In micropolitan and small metropolitan communities, susceptibility is linked to large meat packing plants whose workforce is mostly Hispanic or another ethnoracial group. Outbreaks in these places may exacerbate preexisting racial marginalization and stigma, potentially leading residents of color to be racialized as coronavirus carriers in vulnerable communities with low social capital.^38^

As with any preliminary analysis in a rapidly evolving public health crisis, there are a number of limitations. One is not being able to evaluate the accuracy of the county level susceptibility scale without complete and consistent national data on official infection and mortality rates. A full ex post evaluation will have to wait until sufficient data are collected and verified to understand the full scale and severity of the pandemic. Another limitation is whether counties are the appropriate spatial scale to measure COVID‐19 impacts. In metropolitan areas the proper scale may be municipalities to distinguish between core cities, suburbs, and exurbs that are located within a single county. In the western United States, counties are geographically large and separated by natural barriers. In both cases, county aggregations can result in statistical bias of estimates and hypothesis tests, known as the modifiable areal unit problem.^39^, ^40^ In closing, future research should continue development of a theoretically and empirically rigorous scale measuring susceptibility to pandemics like COVID‐19 to assist in rural health planning.

## Supporting information

Appendix

## Endnotes

i Small independent cities in Virginia with populations under 65,000 were merged back into their respective counties to reduce ACS error margins. Broomfield County in Colorado, newly created in 2003, was disaggregated back into its 4 original counties based on population‐weighted geographic shares. This reduced the number of conterminous counties from 3,109 to 3,079. This permits comparisons with data from 2000 and earlier.

ii The W.E. Upjohn Institute for Employment Research provided unsuppressed employment counts by detailed NAICS industries for each county in the United States. Employment was estimated by applying the Isserman‐Westervelt algorithm to public‐use County Business Patterns (CBP) from the US Census. Estimates for 2017 have been delayed because of changes in CBP reporting by the federal government, so 2016 data is used.

iii Five factors were indicated by the Kaiser‐Guttman rule and Velicer's MAP test, and 6 factors by Horn's parallel analysis. Nursing and elderly care facilities was poorly fitted with low factor loadings and communalities in the 6 factor solution, so an additional factor was extracted to improve fit.

iv Urban influence codes for 2003 instead of 2013 were used to permit comparisons with data from 2000 and earlier, although this was not done in the present analysis.
