# Supplementary material for: Community Susceptibility and Resiliency to COVID‐19 Across the Rural‐Urban Continuum in the United States
Source: J Rural Health. 2020 Jun 16;36(3):446–56. doi: 10.1111/jrh.12477 (PMC7323251; doi:10.1111/jrh.12477)
Supplement: Supplementary file 1 — Appendix [file JRH-36-446-s001.docx]

**Appendix**

**Table A1.** Means of COVID-19 Susceptibility Indicators

|  |  | Metropolitan | | |  | Non-Metropolitan | | |
| --- | --- | --- | --- | --- | --- | --- | --- | --- |
|  |  | (a)  Large  Metro  N=403 | (b)  Midsize  Metro  N=321 | (c)  Small  Metro  N=342 |  | (d)  Micro-  politan  N=665 | (e)  Semi-  Rural  N=803 | Rural  N=545 |
| Population density (sq.mi) |  | 13.77 | 2.95 | 1.47 |  | 0.71 | 0.32 | 0.15 |
| Group quarters (%) |  | 2.27 | 2.80 | 3.49 |  | 3.74 | 3.98 | 3.59 |
| Age 65-84 (%) |  | 13.46 | 14.76 | 14.77 |  | 15.65 | 17.11 | 19.18 |
| Age 85 and older (%) |  | 1.75 | 1.96 | 2.02 |  | 2.19 | 2.41 | 2.87 |
| Elderly & nursing care empl. (10k)^†^ |  | 23.95 | 27.72 | 27.03 |  | 27.55 | 20.39 | 16.85 |
| Cancer mortality (100k) |  | 158.81 | 161.12 | 164.20 |  | 168.38 | 172.08 | 162.49 |
| Cardiovascular mortality (100k) |  | 221.98 | 229.93 | 238.04 |  | 249.02 | 256.96 | 233.54 |
| Lower respiratory mortality (100k) |  | 43.81 | 48.18 | 50.80 |  | 53.99 | 55.58 | 52.13 |
| Diabetes mortality (100k) |  | 20.83 | 23.85 | 24.30 |  | 26.47 | 27.74 | 27.47 |
| Flu and pneumonia mortality (100k) |  | 14.44 | 14.74 | 15.36 |  | 17.45 | 17.43 | 17.26 |
| Meat processing empl. (10k)^†^ |  | 8.51 | 22.93 | 31.02 |  | 57.33 | 48.74 | 29.55 |

*Notes: †=establishment employment.*

**Figure A1.** Scree Plot and Parallel Analysis for N=3,079 Counties in the Conterminous US
